# Boosts in Polarization and Piezoelectric Responses of Lead‐Free Ferroelectrics through Strain‐Enhanced Glassy Coexistent Polars with High Dynamics

**DOI:** 10.1002/advs.202502973

**Published:** 2025-07-11

**Authors:** Liqiang He, Le Zhang, Yating Ran, Zibin Chen, Chuanxin Liang, Yanshuang Hao, Jianwei Li, Zhizhi Xu, Sen Yang, Michael A. Carpenter, Xiaobing Ren, Dong Wang

**Affiliations:** ^1^ Frontier Institute of Science and Technology and School of Physics State Key Laboratory for Mechanical Behavior of Materials and MOE Key Laboratory for Non‐equilibrium Synthesis and Modulation of Condensed Matter Xi'an Jiaotong University Xi'an Shaanxi 710049 P. R. China; ^2^ Department of Earth Sciences University of Cambridge Cambridge CB2 3EQ UK; ^3^ State Key Laboratory of Ultra‐precision Machining Technology and Research Institute for Advanced Manufacturing Department of Industrial and Systems Engineering The Hong Kong Polytechnic University Hong Kong 999077 P. R. China; ^4^ Center for Functional Materials National Institute for Materials Science 1‐2‐1 Sengen, Tsukuba Ibaraki 305‐0047 Japan

**Keywords:** glassy transitions, lead‐free ferroelectrics, phase field simulations, polar dynamics, strain engineering

## Abstract

The miniaturization of domain size via point‐defect modification has emerged as an effective strategy for optimizing piezoelectric properties in lead‐free ferroelectrics, such as achieving large piezoelectric constants and slim‐hysteresis electrostrain through polymorphic nanodomain design. However, this approach is not consistently reliable due to the uncontrollable deceleration of domain wall motion and polarization rotation (i.e., polar dynamics) caused by the random local fields around doping sites. In this work, an innovative strategy is proposed to enhance the polarization and electrostrain responses of nanodomain‐patterned ferroelectrics through the design of a strain‐enhanced glassy coexistent polar state (SGP), which can be established by incorporating large‐radius cation Hf^4+^ into (Ba_0.84_Ca_0.16_)_0.985_Bi_0.01_(Ti_0.9_Zr_0.07_Sn_0.03_)O_3_ compositions. The strain‐enhanced crossover state between the neighboring glassy tetragonal and orthorhombic domains greatly facilitates overall polar dynamics. This is evidenced by the large dielectric figure of merit *ε*_r_/tan *δ* ≈ 9.9×10^5^ with thermal stability up to 23 K, surpassing most of reported lead‐free ferroelectric ceramics. Importantly, compared to the initial matrix, significant improvements of 20.8% and 34.2% in maximum polarization and electrostrain amplitude are achieved, while maintaining a minimal polarization/strain hysteresis (≈5%). This work would pave a novel paradigm for designing superior functional ferroics by optimizing the domain dynamics.

## Graphical abstract

An effective strategy by constructing strain‐enhanced glassy coexistent polars near ferroelectric tricritical point to simultaneously improve polarization response and reduce energy loss is uncovered in (Ba_0.84_Ca_0.16_)_0.985_Bi_0.01_(Ti_0.9_Zr_0.07_Sn_0.03_)_1‐_ *_x_*_/100_Hf*_x_*_/100_O_3_ (*x*Hf) system and verified by phase‐field simulations with supporting high resolution transmission electron microscopy.

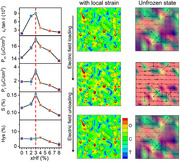

## 1 Introduction

Nano‐sized domain configuration in ferroelectrics, e.g. hierarchy nanostructure at morphotropic phase boundary, can contribute to the superior piezoelectric constants *d*_33_ due to the lowered energy barrier of polarization rotations between differently oriented polars.^[^ ^1^, ^2^, ^3^ ^]^ In comparison, relaxor ferroelectrics with polar nano regions randomly embedded in the paraelectric matrix hold the great potential in various electronic devices, e.g., pulsed energy generator, electromechanical actuator and ultrasonic transducers, due to the low hysteresis, thermally stable polarization and electrostrain responses.^[^ ^4^, ^5^, ^6^, ^7^ ^]^ However, there exists much concern for the drawbacks in the classical nanodomain structures established through the widely‐used doping strategies. For example, cation vacancies and oxygen vacancies caused by aliovalent doping (donor or acceptor, respectively) in a ABO_3_ ferroelectric system could disrupt the formation of long‐range‐order ferroelectric domains and lead to the formation of relaxor ferroelectrics, but also impede the domain wall motion, resulting in the sharply decreased amplitude of related polarization *P* and electrostrain *S*.^[^ ^5^, ^8^ ^]^

In recent years, there appears a novel strategy by Zhang et al., namely glassy ferroelectrics exhibiting high‐density coexistent multiphase nanodomains for superior electromechanical coupling due to the easier domain wall motion and polarization rotations.^[^ ^9^ ^]^ Such kind of ferroelectric nanostructures can be obtained by diffusing conventional ferroelectric transitions through aliovalent doping.^[^ ^9^, ^10^ ^]^ Unfortunately, this strategy does not work well in numerous ferroelectric systems due to the uncontrollable pinning effect from interaction of random vacancies with domain walls, thus facilitating the transformation of ferroelectrics to dispreferred non‐ergodic relaxor ferroelectrics rather than unfrozen glassy ferroelectric state even though a tiny amount of dopants is incorporated in many cases.^[^ ^11^, ^12^ ^]^ For example, the introduction of a little second components such as Sr(Sc_0.5_Nb_0.5_))O_3_, La(Mg_0.5_Ti_0.5_)O_3_, etc., make ferroelectric state of Bi_0.5_Na_0.5_TiO_3_‐based ceramics quickly transform into relaxor ferroelectric state, resulting in the sharp decrease of maximum polarization.^[^ ^13^, ^14^, ^15^, ^16^ ^]^ And BiFeO_3_‐BaTiO_3_ systems doped by a tiny amount of Sb, LiNbO_3_, NaNbO_3_ and so on, sharply miniaturize maze‐like domain structures, leading to the rapid deterioration of electrostrain and converse piezoelectric coefficient.^[^ ^17^, ^18^, ^19^ ^]^ Therefore, it remains an urgent demand to deeply understand the nature and accurate optimization process of ferroelectric nanostructures to reach the formation of unfrozen glassy ferroelectrics with adequate ergodic high‐density multiphase nanodomains.

In general, the characteristics of glassy ferroelectrics are similar to the strain glass in ferroelastics and spin glass in the ferromagnets, including frequency dispersion following the Vogel‐Fulcher law, ergodic‐nonergodic transition, freezing temperature for slow‐down dynamics and so on.^[^ ^20^ ^]^ Unfortunately, the theoretical breakthrough is still lacking to boost up associated electromechanical properties from the perspective of modulating the polar distribution and polar dynamics of glassy ferroelectric nanostructures. And the superior polarization responses under an external electric field, due to existing polymorphic nanodomains, is crucial for piezoelectric, energy storage, and electrocaloric properties.^[^ ^2^, ^21^, ^22^, ^23^, ^24^, ^25^, ^26^ ^]^ For example, the use of polymorphic nanodomain design in ferroelectric systems can enhance the energy storage density due to the enhanced breakdown strength rather than manipulating the polar dynamics from the intrinsic physical nature.^[^ ^5^, ^13^, ^21^ ^]^ Additionally, the relaxor‐like slush polar coexistence can achieve large piezoelectric constant in modified K_0.5_Na_0.5_NbO_3_‐based ceramics due to the increased density of domain boundaries rather than optimizing the mobility of coexistent nanodomains.^[^ ^2^, ^27^, ^28^ ^]^

It is acknowledged that the curvature of the free‐energy landscape and can be decided by the second derivative (∂^2^G/∂P^2^) in thermal equations, i.e., a small curvature (referred to as a flat energy barrier) corresponds to a high mobility of domain walls.^[^ ^29^ ^]^ The dielectric permittivity, a fundamental indicator of polar dynamics, can be measured by the polarization change under the alternating (AC) electric field. The dielectric permittivity (*ε_r_*) equals ∂*P*/∂*E*, which quantifies the ease degree for the polarizing of ferroelectric materials under applied electric field, reflecting its electric field susceptibility. And the dielectric loss (tan *δ*) can be calculated by the integral area enclosed by the polarization‐electric field (*P*–*E*) loop,^[^ ^30^ ^]^ reflecting the extent to which the materials dissipate electrical energy under applied electric field. The high dielectric permittivity with low loss is extremely important for the optimization of associate polarization and piezoelectric performance under applied electric field. Therefore, the materials with high dielectric permittivity and low dielectric loss have attracted much attention in the associated electronic devices. Theoretically, the peaks of *ε*_*r*_ can be secured by locating the material composition and temperature at the morphotropic phase boundary (MPB) and the dielectric values can be maximized at the tricritical point (the crossover between first and second order phase transition) due to the most flattened energy profile of ferroelectric phases.^[^ ^31^, ^32^ ^]^ In fact, the as‐designed phase convergence region with tricritical characteristics exhibit much complex polar distributions, generating large loss under driving field possibly due to the jamming of domain wall motion and various polar rotations. For example, the tricritical point of Sn‐doped BaTiO_3_ ceramics was proved as a coexistence of quadruple states (Cubic (C), tetragonal (T), orthorhombic (O), rhombohedral (R)),^[^ ^32^, ^33^ ^]^ the loss of which is higher than neighboring compositions with coexistence of less phases although the larger *ε*_*r*_ is reached. The rotation barrier for T–O transitions is lower than that of O–R transitions due to the existence of group‐subgroup relation between T and O phases.^[^ ^34^ ^]^ It can be conjectured that the energy loss of tricritical point could be further diminished if the number of polar states was decreased, especially the coexistence of C, T and O phases.

In this work, the high polar dynamics with low energy loss are realized in phase‐field simulation by constructing the strain‐enhanced glassy coexistent polar state (SGP) with only three states, i.e., cubic, glassy tetragonal, and orthorhombic domains. Experimentally, such a polar distribution state is verified in (Ba_0.84_Ca_0.16_)_0.985_Bi_0.01_(Ti_0.9_Zr_0.07_Sn_0.03_)_1‐_ *_x_*_/100_Hf*_x_*_/100_O_3_ (abbreviated as *x*Hf) ceramics by using high resolution transmission electron microscopy. Importantly, there appears some strain‐enhanced crossover state between T and O glassy nanodomains. Such a microstructure configuration enables the significant boosts of *ε_r_* with low dielectric loss, corresponding to the high mobility of domain wall motion and polarization rotations, perfectly resulting in desired enhancements of maximum polarization and electrostrain amplitude as anticipated. In‐depth physical understanding for the phenomena has also been addressed through employing phase‐field simulations with the inputs of local strain field from actual glassy nanostructure configuration.

## 2 Results and Discussions

**Figure 1:**
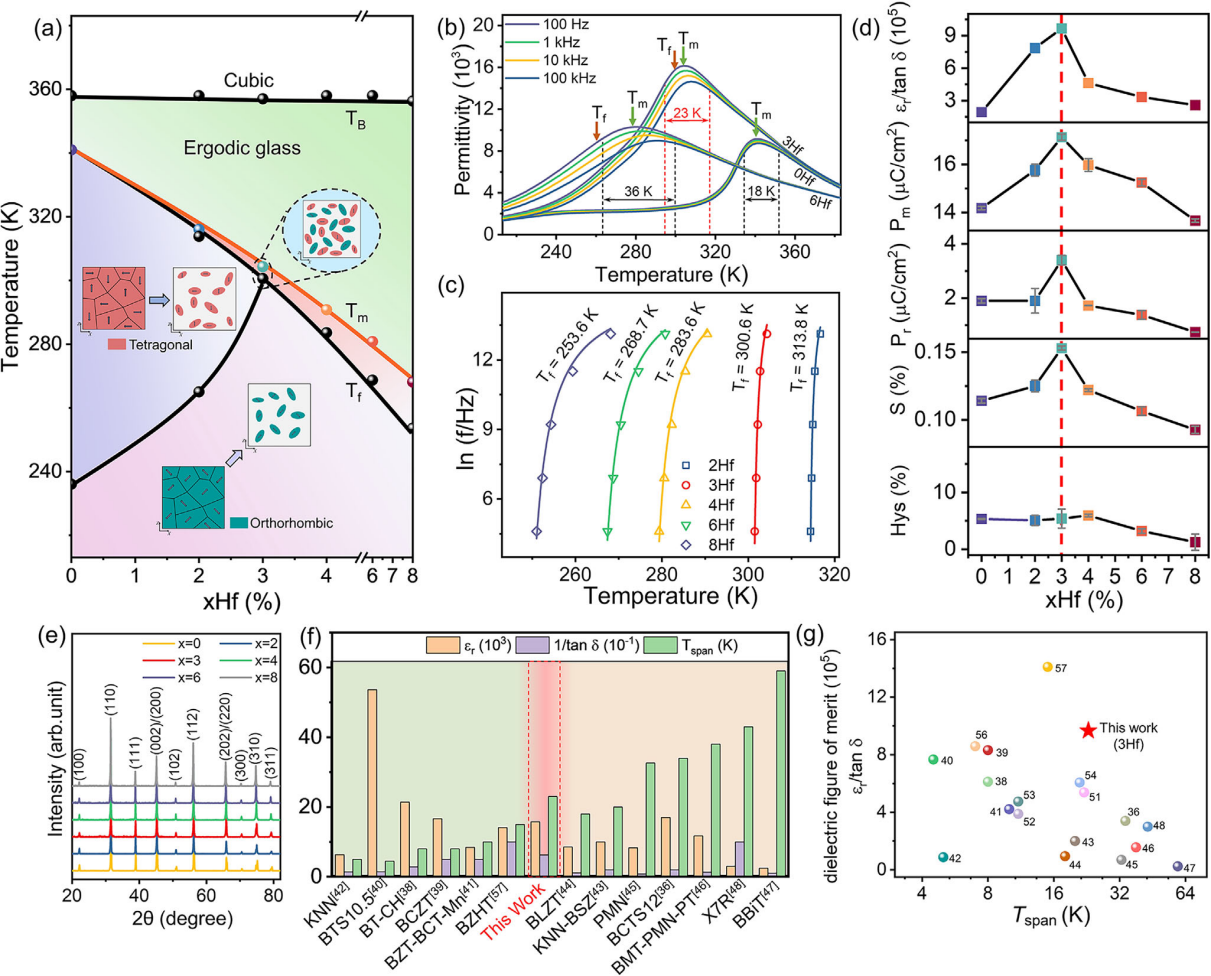
a) The temperature‐composition phase diagram of *x*Hf ceramic system. b) The permittivity (*ε*_*r*_)‐ temperature (*T*) curves of 0Hf, 3Hf and 6Hf with *T*_span_ under different test frequencies. c) *T*_m_ as a function of the frequency, fitted by the Vogel‐Fulcher relation, showing the corresponding *T*_f_. d) Composition‐dependent *ε*_*r*_/tan *δ*, the maximum polarization (*P*_max_), the remnant polarization (*P*_r_), electrostrain (*S*) and strain hysteresis (Hys) along the *T*_m_ line. e) XRD patterns of *x*Hf ceramics in the 2θ range of 20–80°. f) Comparison of dielectric permittivity (*ε*_*r*_, 10^3^), quality factor (1/tan *δ* 10^−1^) and *T*_span_ of 3Hf with other reported representative ferroelectric and relaxor ceramics. g) Comparison of dielectric figure of merit (*ε*_*r*_/tan *δ*) versus *T*_span_ of 3Hf with reported representative ferroelectric and relaxor ceramics.

**Figure 2:**
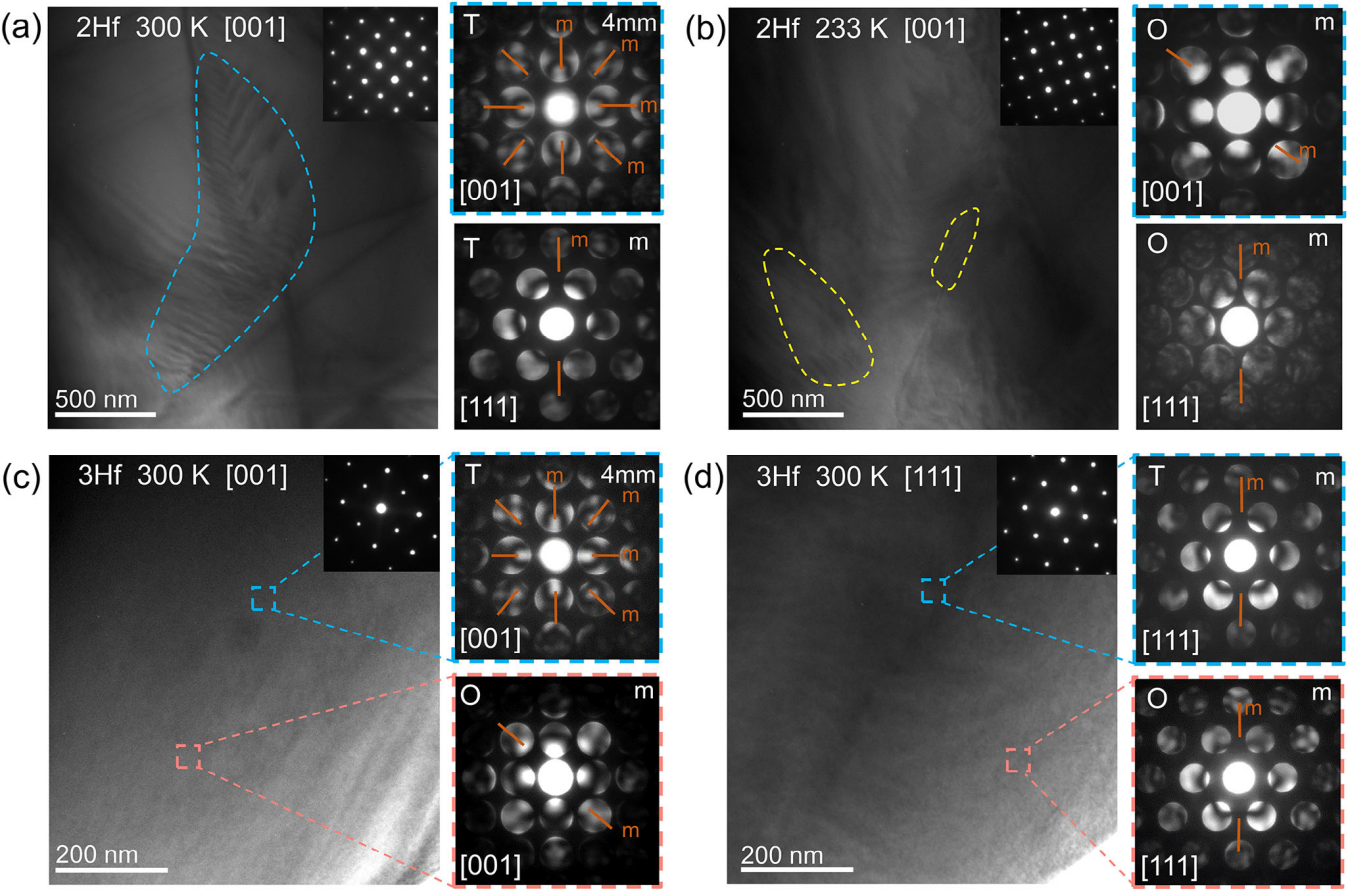
a,b) In situ domain structures evolution along [001] zone axis and associated convergent beam electron diffraction (CBED) patterns along [001] and [111] zone axes in the selected region of 2Hf at 300 K and 233 K, respectively. c,d) Domain structures and CBED patterns of 3Hf at 300 K along [001] and[111] zone axes.

**Figure 3:**
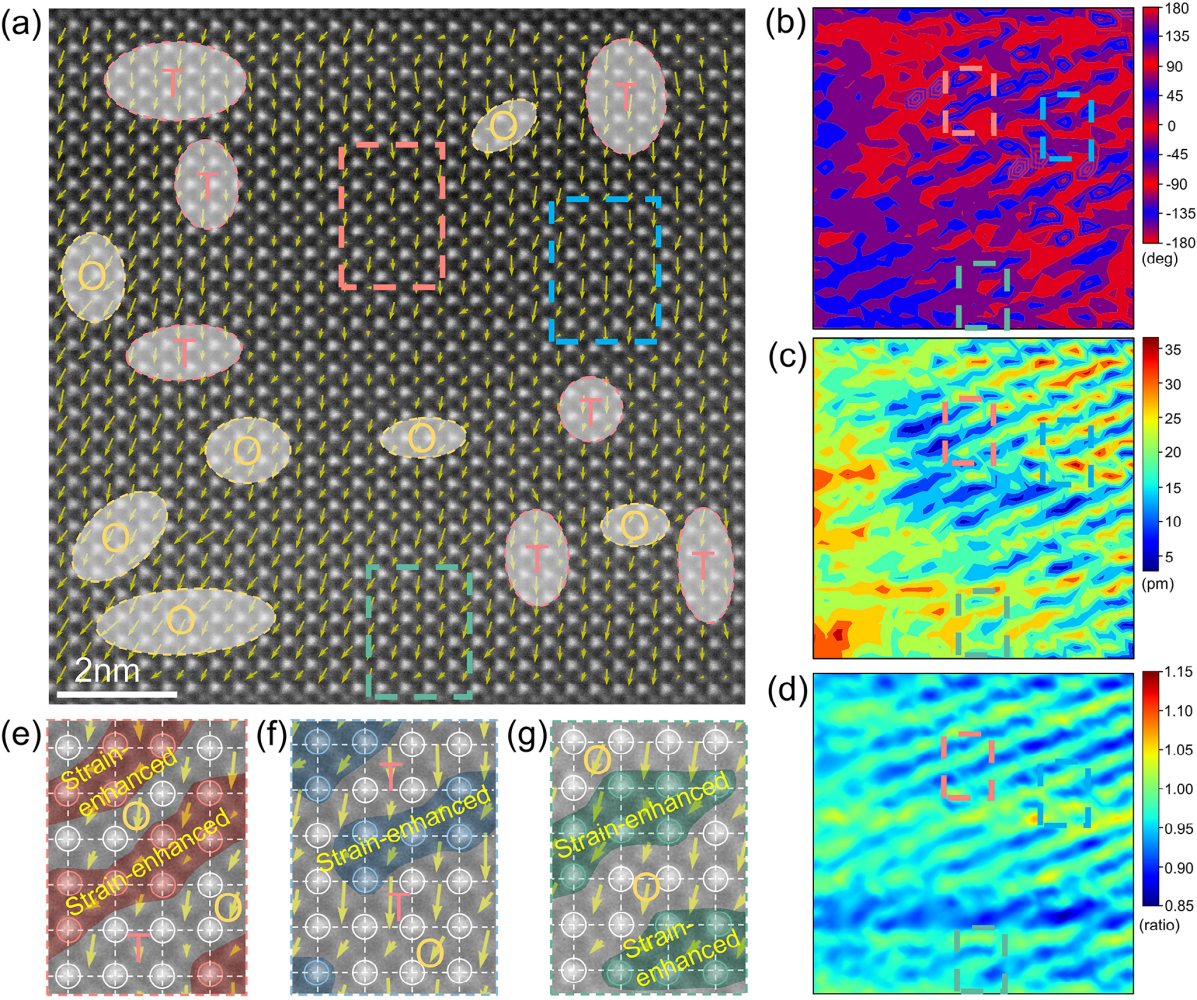
a) Atomic‐resolution HAADF‐STEM polarization vector image along [001] beam incidence. b) Polarization angle mapping. c) Polarization magnitude mapping. d) The ratio of space mapping of B‐site, e–g) Enlarged images showing strain‐enhanced glassy coexistent polars (SGP) with large lattice strain (deep red, blue, green) and low polarization magnitude (short arrows) among nano‐scaled tetragonal (T) and Orthorhombic (O) phases.

**Figure** **1**a presents the phase diagram of *x*Hf, giving the information of Burns temperature (*T*_B_), the temperature of maximum permittivity (*T*_m_), the freezing temperature (*T*_f_),^[^ ^35^ ^]^ and the schematic evolution of ferroelectric structures, determined by the dielectric permittivity *ε_r_* versus Temperature *T* profiles (see Figure 1b, Figures S1, S2, Supporting Information), convergent beam electron diffraction (CBED, **Figure** **2**), and high‐angle annular dark‐field scanning transmission electron microscopy image with polarization vector mapping (HAADF‐STEM, **Figure** **3**). It is clear that there is no rhombohedral phase in *x*Hf system designed here. With the increase of Hf dopant, the *T*_f_ and the temperature of T–O phase transition lines are inclined to form a C–T–O convergent region near 3Hf. The *ε_r_–T* curves in Figure 1b demonstrate an increase in frequency dispersion, a decreased *T*_m_, and the variation of dielectric values with increasing Hf concentration. Importantly, the maximum permittivity with lowest loss appears at *T*_m_ of 3Hf simultaneously, as illustrated in Figures S3, S4 (Supporting Information). The highest average dielectric permittivity (*ε_r_*) of approximately 15800 with a low average dielectric loss (tan *δ*) of around 0.016 at 100 Hz is achieved in a temperature range of 294–317 K (*T*_span_ ≈23 K) due to the tricritical feature of C–T–O phase coexistence region, suggesting superior polar dynamics with thermal stability in 3Hf as expected (i.e., mobile polar motion with minimal energy loss). *T*_span_ is defined as the temperature range where permittivity exceeds 90% of the maximum.^[^ ^36^ ^]^ Thus, 3Hf exhibits large comprehensive dielectric properties when taking *ε_r_*, quality factor 1/tan *δ* and temperature stability (see Figure 1f) into consideration. To quantify the ease of polar motion relative to energy loss, the dielectric figure of merit *ε*_*r*_/tan *δ* is employed. Figure 1f,g and Table S1 (Supporting Information) show the comparison of *ε*_*r*_/tan *δ* versus *T*_span,_ where the thermally stable *ε*_r_/tan *δ* value is approximately 9.9×10^5^ of 3Hf ceramics. 3Hf ceramics outperform most of reported Hf‐doped BaTiO_3_‐based ceramic systems when taking *ε*_*r*_, tan *δ*, dielectric figure of merit (*ε*_*r*_/tan *δ*) and *T*_span_ into consideration. Although the Ba(Hf_0.08_Ti_0.92_)O_3,_ BHT10, BT‐CH cases exceed 3Hf in *ε_r,_* their tan *δ* and *T*_span_ are much inferior to 3Hf.^[^ ^37^, ^49^, ^50^, ^51^, ^52^, ^53^, ^54^, ^55^, ^56^, ^57^ ^]^ In addition, it surpasses most of other reported representative ferroelectric ceramics, relaxor ceramics, and commercial X7R materials when taking both *ε*_*r*_/tan*δ* and *T*_span_ into consideration.^[^ ^36^, ^37^, ^38^, ^39^, ^40^, ^41^, ^42^, ^43^, ^44^, ^45^, ^46^, ^47^, ^48^, ^49^, ^50^, ^51^, ^52^, ^53^, ^54^, ^55^, ^56^, ^57^ ^]^

The composition dependence of *ε*_*r*_/tan *δ*, *P* and *S* of *x*Hf ceramics at *T*_m_ is summarized in Figure 1d and the detailed *P*–*E* and electrostrain‐electric field *S*–*E* loops are presented in Figure S5 (Supporting Information). In the various compositions of *x*Hf, 3Hf with C–T–O covergence region exhibits the highest average *ε*_*r*_/tan *δ*, maximum polarizations *P*_max_, remnant polarization *P*_r_, and coupled electrostrain *S* with low strain hysteresis (Hys) near *T*_m_. Compared with 0Hf, the *P*_max_ and *S* of 3Hf increase by 20.8% and 34.2% respectively while maintaining a slim hysteresis (≈5%). It indicates that the superior polar dynamics of 3Hf directly lead to significant optimization of polarization and coupled electrostrain properties. With further increase of Hf to *x* = 4–8, no C–T–O coexistent state persists and the great pinning effect from high‐level Hf will degrade the polarization and electrostrain amplitude, evidenced by the rapid increased dielectric loss in Figure S3 (Supporting Information).

Figure 1e shows X‐ray diffraction (XRD) patterns in the 2*θ* range of 20–80°, confirming the purity of perovskite structures. And Figure S6 (Supporting Information) reveals the global structural transformation from a tetragonal of 0Hf to an average cubic structure near 3Hf. The (002) and (220) peaks continuously shift to low diffraction angles with Hf doping because Hf cation possesses a larger ionic radius (0.71 Å) than the average radius (0.69 Å) of B‐site in 0Hf matrix.^[^ ^58^ ^]^ In addition, Rietveld refinement results for the XRD data of *x*Hf ceramics in Figure S7 (Supporting Information) indicates that the coexistent C–T–O phases appear in 3Hf at room temperature. With increasing Hf concentration, the percentage of C phase gradually increases, while the T phase decreases rapidly from 0Hf to 3Hf, and the O phase gradually decreases from 3Hf to 8Hf. The microstructural evolutions are well consistent with the established phase diagram in Figure 1a. As shown in Figure S8 (Supporting Information), the average grain size sharply decreases from 14.08 µm of 0Hf to 11.51 µm of 2Hf. With additional increase of Hf concentration, the average grain size remains stable between 11.5 and 11.3 µm although there are significant changes in dielectric responses, suggesting the negligible impact of grain size on dielectric properties within 2–8Hf. In addition, the scanning electron microscopy‐ energy dispersive X‐ray spectroscopy (SEM‐EDX) mapping in Figure S9 (Supporting Information) shows that all elements are uniformly distributed in all *x*Hf ceramics without signs of agglomeration.

The frequency dispersion of *T*_m_ in *x*Hf (*x* = 2, 3, 4, 6 and 8) are well fitted by Vogel‐Fulcher relation, as shown in Figure 1c, confirming the formation of glassy ferroelectrics with nano‐sized domain configurations.^[^ ^35^ ^]^ Additionally, the slight divergence of *T*_m_ and *T*_f_ starting from 2–3Hf suggests that some ergodic polar nanodomains commence to appear. More introduction of Hf like *x* = 4, 6 and 8 will intensify the divergence between *T*_m_ and *T*_f_. From 2Hf to 8Hf, the activation energy *E*_a_ increases from 0.61‐0.96 meV of 2–3Hf to 3.36‐3.82 meV of 4–8Hf, indicative of deteriorated mobility of nanodomains for glassy ferroelectric state with Hf doping (see Table S2, Supporting Information). And the high activation energy of 4–8Hf compositions impede the transition and rotation of nano polar state below *T*_f_, resulting in inferior polarization and piezoelectric outputs. In comparison, the *E*_a_ of 3Hf is significantly smaller in comparison with 4, 6 and 8Hf and close to the case of 2Hf, suggesting the nearly unfrozen glassy state even below *T*_f_.

More evidence and local symmetry information for nano‐sized microstructure of glassy ferroelectric state is given in transmission electron microscopy observations. Figure 2a shows the appearance of ferroelectric microdomains with a tiny amount of nanodomain on the side in 2Hf at 300 K. The CBED results suggest the T symmetry of 2Hf at 300 K. Upon cooling to 233 K, the long‐range‐order ferroelectric domains of 2Hf becomes indistinct and local symmetry becomes O as shown in Figure 2b. In comparison, the 3Hf does not exhibit any long‐range‐order ferroelectric domains at 300 K. The CBED patterns of 3Hf captured from different regions and zone axes ([001] in Figure 2c and [111] in Figure 2d) illustrate two coexistent types of local symmetry for polar nano regions: T and O at 300 K,^[^ ^59^ ^]^ suggesting the existence of T–O coexistent glassy polar states at 3Hf near room temperature. Such a slightly frozen coexistent glassy state with low activation energy corresponds to the ultralow energy barrier for polarization rotation and facilitates the formation of a well‐aligned polar state under external electric field. In 4Hf with high *E*_a_, only a glassy state with O local symmetry exists at 300 K (see Figure S10, Supporting Information). In addition, as shown in CBED results of 3–4Hf (Figure S10, Supporting Information), there is only O local symmetry even cooling down to 233 K for both two samples, solidifying the evidence for the absence of extra phase transitions below T*_f_* of 3–4Hf.

Integrated differential phase contrast scanning transmission electron microscopy (iDPC‐STEM) observations are employed to further disclose the microstructure origin of 3Hf.^[^ ^2^, ^60^ ^]^ The related polarization angle mapping analysis along [110] zone axis for 3Hf in Figure S11 (Supporting Information) displays that there exist T and O nanoregions, verifying the absence of the R phase. And the localized energy‐dispersive X‐ray spectroscopy mappings of 3Hf along [110] zone axis further certifies the chemical homogeneity, as shown in Figure S12 (Supporting Information). HAADF‐STEM observations in Figure 3a also indicate that there exists the cubic components with nearly‐zero polarization magnitude in 3Hf besides the existence of randomly distributed T and O glasses, demonstrating the 3Hf composition indeed exhibit the coexistent C‐T‐O glasses.^[^ ^61^, ^62^ ^]^ It enables the largest dielectric constants appear in *ε*_*r*_ –*T* results. More importantly, during the large‐size stripe region (≈5–10 nm) with the intermediate polarization angle in Figure 3b, small polarization magnitude in Figure 3c, and a large localized *c*/*a* ratio of B‐site in Figure 3d are formed between the nano‐scaled T and O phases. For normal ferroelectric structure, the large *c*/*a* ratio always accompanies with the large spontaneous polarization. Such a different performance of microstructure in 3Hf (large *c*/*a*, low *P*) is ascribed to the influence of local strain heterogeneity (Hf^4+^→Zr^4+^) on the glassy coexistent polar state. Figure 3e–g (enlarged figures of the related dashed boxes) further emphasize these features, illustrating a crossover zone characterized by a pronounced local strain field among the nano‐scaled T and O phases. Away from tricritical state of 3Hf, stable ferroelectric phase in 2Hf and deeply frozen glassy ferroelectric state in 4–8Hf will both go against the formation of strain‐enhanced glassy coexistent polars (SGP) with high dynamics.

**Figure 4:**
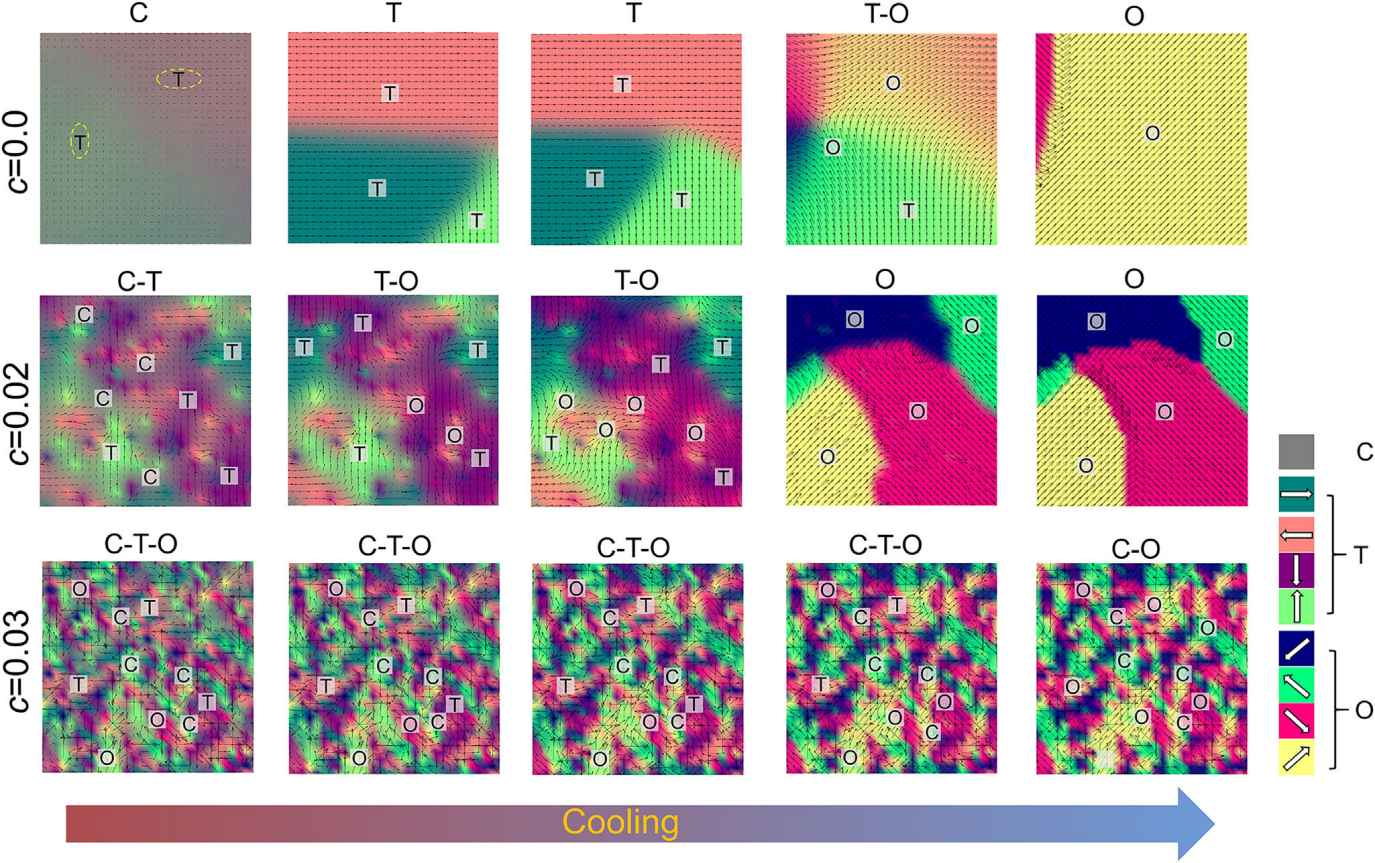
Calculated evolution of ferroelectric domain structures at *c* = 0, 0.02, and 0.03 upon cooling. *c* is the concentration of local strain in accordance with Hf‐doped concentration.

To uncover the effect of the extra local strain field of crossover on the glassy coexistent microstructure and electrical properties, phase field simulation is employed in SGP composition.^[^ ^63^, ^64^ ^]^ The tensors of lattice strain (*ε* ^local^) resulted from B‐site ion displacement shown in Figure 3d and Figure S13 (Supporting Information), are extracted as the input for subsequent phase field modelling to make sure the simulation accuracy and reliability.^[^ ^65^, ^66^, ^67^, ^68^ ^]^ As shown in **Figure** **4**, a pure ferroelectric solid solution exhibiting a phase transformation from cubic to tetragonal to orthorhombic upon cooling, is constructed firstly, where *c* is the concentration of local strain in accordance with Hf‐doped concentration. The introduction of a large‐radius Hf dopant that can induce local strain heterogeneity and stabilize the O state, resulting in the appearance of T–O coexistent miniaturized ferroelectric domains at *c* = 0.02. When the *c* is further increased to *x* = 0.03, the C–T–O coexistence region can be achieved, consistent with the static microstructure pattern of SGP observed in 3Hf. Additionally, C–T–O coexistent ferroelectric nanodomain of SGP at *x* = 0.03 can persist over a wide temperature range.

**Figure 5:**
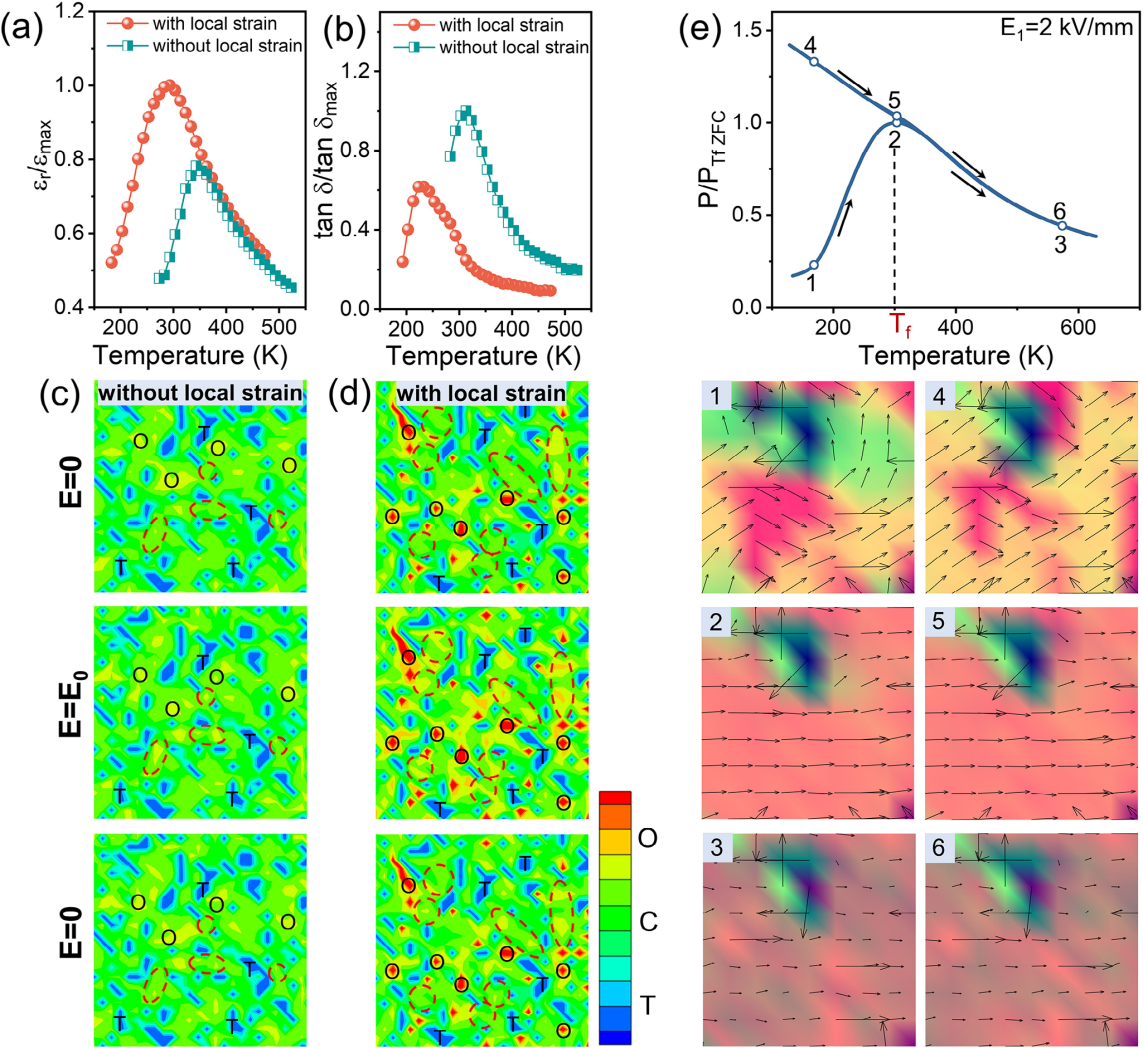
Calculated temperature‐dependent normalized a) dielectric permittivity *ε*_*r*_ and b) dielectric loss tan *δ* without or with local strain field of SGP by applying a sinusoidal electric field. Electric‐field‐induced microstructural evolution of SGP c) without and d) with local strain field near the *T*_m_. e) The calculated zero‐field‐cooling (ZFC)/field‐cooling (FC) curves and the microstructural evolution of ferroelectric domains of SGP with local strain field.

The temperature‐dependent dielectric permittivity and loss are calculated in *c* = 0.03 when a small sinusoidal electric field (*E*_0_ = 0.2 kV mm^−1^, much lower than the coercive field *E*_C_) is applied, aiming to investigate the impact of local strain field of crossover on dielectric responses and associated energy loss of C–T–O glassy coexistent polar state. **Figure** **5**a,b illustrates that introducing the local strain field to the coexistent glasses results in an extra 25.0% increase in peak dielectric permittivity and a 38.8% further decrease in dielectric loss, compared to those without local strain field. The associated microstructural evolution upon the application and withdrawal of the electric field *E*_0_ is reflected by the symmetry contours in Figure 5c,d, where much more field‐induced O phase shown in “red circles” are observed under the condition with local strain field rather than that without local strain field. The simulation results reveal that the involvement of local strain field facilitates the formation, growth and reversibility of the O phase, leading to the higher polar dynamic of C–T–O glassy coexistent polar state with lower energy loss.

Figure 5e illustrates the variation in ergodicity (another characteristic of glassy ferroelectrics) and the microstructural evolution of such SGP through zero‐field‐cooling (ZFC) and field‐cooling (FC) protocols under a significant [100] DC bias field (*E*_1_ = 2 kV mm^−1^, higher than *E*_C_).^[^ ^69^ ^]^ The details of ZFC/FC measurement are described in Figure S14 (Supporting Information). The SGP composition (*c* = 0.03) exhibit the history dependence, i.e., non‐ergodicity below the freezing temperature *T*_f_, as evidenced by the deviation of ZFC (1→2→3) and FC (4→5→6) curves. The random domain structure of the SGP will be frozen when temperature is set below *T*_f_, resulting in different domain patterns and low polarization (see Points 1). Upon heating to *T*_f_ (Points 2 and 5), the disordered configuration of T and O nanodomains can thaw and subsequently align to the almost same domain patterns along the direction of applied electric field due to the achievement of high polar dynamics through defreezing process of the glassy state. Upon further heating, the ferroelectric phases for both ZFC and FC conditions will depolarize, resulting in the reduced polarization value at Points 3 and 6. Thus, the experimental *T*_span_ for 3Hf around *T*_f_ doesn't only allow the occurrence of high polar dynamics but also impedes the depolarization process. It will enable the large polarization and coupled strain possibly to appear under the high electric field.

**Figure 6:**
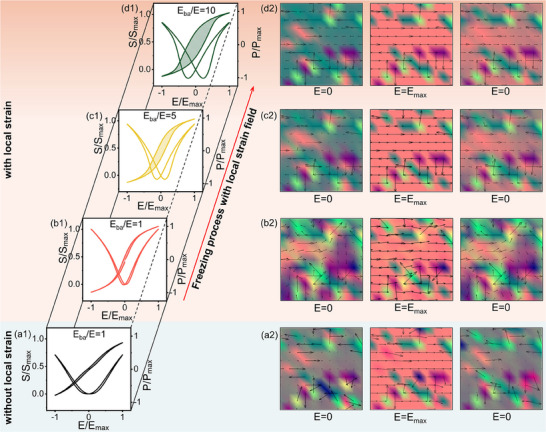
Calculated freezing process of T–O coexistent glassy polar state under the applied electric field along [100] direction after the introduction of local strain field. *S*_max_, *P*_max_ and *E*_max_ represent the maximum value of electrostrain, polarization and applied electric field. Normalized *S*–*E*, *P*–*E* loops and the corresponding microstructural evolution of systems (a) without local strain field at *E*_ba_/*E* = 1 and with local strain field at b) *E*_ba_/*E* = 1, c) *E*_ba_/*E* = 5, and d) *E*_ba_/*E* = 10.

**Figure** **6** shows the calculated *P*–*E* and *S*–*E* loops of *x* = 0.03 composition under the electric field (maximum electric field *E*_max_ = 5 kV mm^−1^, much higher than *E*_C_), accompanied by the relevant microstructural evolution with varying dynamic conditions of polar rotation, where *E*_ba_/*E* represents the energy barrier of polar rotation in simulation. Figure 6a demonstrates the *P*–*E* and *S*–*E* loops and microstructural evolution of C‐T‐O glassy coexistence state under the condition of *E*_ba_/*E* = 1 and no local strain field applied. Even though the energy barrier for T and O polarization rotation is low and a minimal hysteresis is achieved, the overall *P*_max_ and coupled electrostrain are relatively low when the electric field is applied. Interestingly, the composition of *c* = 0.03 with local strain field can strongly enhance *P*_max_ and coupled electrostrain *S* (see Figure 6b), alternative from the *x* = 0.03 case without local strain field (see Figure S15, Supporting Information) due to the large polarization enhancement (Δ*P*) arising from the local strain field (see Figure S16, Supporting Information). In addition, the temperature‐dependent *P–E* and *S–E* loops of both cases “with local strain” and “without local strain” are calculated from 283 to 323 K (see Figure S17a,b, Supporting Information). As shown in Figure S17c (Supporting Information), it is clear that the introduction of local strain field leads to the enhanced thermal stability of maximum polarization and electrostrain. The hysteresis of polarization and electrostrain represents the energy dissipation, shown by the enclosed areas in *P*–*E* and *S–E* loops. It is worth noting that the coexistent nanodomains can fully return to its initial state upon removal of the electric field near *T*_f_ either with or without local strain field (see Figure 6a2,b2), leading to a slim hysteresis. But as the temperature decreases from *T*_f_, the increased *E*_ba_/*E* by freezing process causes the more difficult polar recoverability (see Figure 6c2,d2), leading to the obviously enhanced hysteresis from *E*_ba_/*E* = 5 (see Figure 6c1) to *E*_ba_/*E* = 10 (see Figure 6d1). And the corresponding *P*_max_ and *S* also gradually decrease. Therefore, the SGP state near *T*_f_ rendering large Δ*P* and ultralow energy barrier in polar rotations is crucial for achieving substantial dielectric, polarization, and strain responses while maintaining the low energy loss.

## 3 Conclusion

In summary, through experimental investigation and systematic theoretical calculations, we have elucidated the high polar dynamics of strain‐enhanced glassy coexistent polars (SGP) near triple point with low activation energy and make clear the physical influence on the ferroelectric and piezoelectric properties. The local strain field arising from lattice distortion can push forward the formation of O polar nano regions in glassy coexistent state and promote the domain recoverability and thermal stability, giving a pathway for optimizing the dielectric response, polarization amplitude and coupled electrostrain values. Experimentally, the presence of SGP in Hf‐doped BaTiO_3_‐based lead‐free ferroelectrics facilitates the significant enhancements in maximum polarization ≈20.8% and strain outputs ≈34.2% while maintaining low energy dissipation ≈5% due to the achievement of superior polar dynamics indicated as the large reported dielectric figure of merit *ε*_*r*_/tan*δ* ≈9.9×10^5^. Our work proposes an effective strategy for achieving high field‐induced energy outputs with adequate thermal stability while minimizing energy dissipation in ferroic materials by implanting strain engineering into coexistent glassy state.

## 4 Experimental Section

### Sample Fabrication

(Ba_0.84_Ca_0.16_)_0.985_Bi_0.01_(Ti_0.9_Zr_0.07_Sn_0.03_)_1‐_ *_x_*_/100_Hf*_x_*_/100_O_3_ (abbreviated as *x*Hf) ceramics were synthesized by the solid‐state reaction method. The starting chemical powders are BaZrO_3_ (99%, Alfa Aesar), BaCO_3_ (99.8%, Alfa Aesar), TiO_2_ (99.9%, Alfa Aesar), Bi_2_O_3_ (99.9%, Alfa Aesar), CaCO_3_ (99.9%, Alfa Aesar), SnO_2_ (99.9% Alfa Aesar) and HfO_2_ (99%, Alfa Aesar). The calcining was performed at 1573 K for 3 h and the sintering was done at 1723 K for 3 h.

### Experimental Characterization

The polarization/electrostrain‐electric field (*P*/*S*–*E*) loops were measured by Premier II ferroelectric test systems with a laser interferential vibrometer and an aixTEMP 5A system. The temperature‐dependent dielectric permittivity was collected by a HIOKI3532 LCR meter with a heating rate of 2 K min^−1^. X‐ray diffraction (XRD) patterns were collected by Shimadzu XRD7000. Convergent beam electron diffraction (CBED) measurements were performed by JEOL‐2100F transmission electron microscope with a cooling system. The morphology images of the samples were obtained by scanning electron microscopy (Sigma 360) with energy dispersive X‐ray spectroscopy detector. Scanning transmission electron microscopy (STEM) observations were carried out using a double aberration‐corrected Thermo Scientific Spectra 300 (S)TEM operated at 300 kV with a convergence semi‐angle of 24.4 mrad with a high‐angle annular dark‐field (HAADF), an integrated differential phase contrast (iDPC) and energy‐dispersive X‐ray spectroscopy detectors.

### Phase Field Simulation

The domain structure in the system is described by a spontaneous polarization **P** (*P*_1_, *P*_2_, *P*_3_). The total free energy of the system can be described as following:^[^ ^70^ ^]^

(1) $$F=\int_{V}\left(f_{\mathrm{lan}}+f_{\mathrm{grad}}+f_{\mathrm{elas}+\mathrm{localelas}}+f_{\mathrm{elec}}+f_{\mathrm{localelec}}\right)dV$$

where the landau free energy is written in terms of *P* as:

(2) $$\begin{array}{ccc} f_{\mathrm{lan}} & = & A_{1}\left(P_{1}^{2}+P_{2}^{2}+P_{3}^{2}\right)+A_{11}\left(P_{1}^{4}+P_{2}^{4}+P_{3}^{4}\right)+A_{12}\left(P_{1}^{2}P_{2}^{2}+P_{2}^{2}P_{3}^{2}+P_{1}^{2}P_{3}^{2}\right)\\ & & +A_{111}\left(P_{1}^{6}+P_{2}^{6}+P_{3}^{6}\right)+A_{112}(P_{1}^{4}P_{2}^{2}+P_{2}^{4}P_{3}^{2}+P_{1}^{4}P_{3}^{2}+P_{1}^{2}P_{2}^{4}+P_{2}^{2}P_{3}^{4}\\ & & +P_{1}^{2}P_{3}^{4})+A_{113}\left(P_{1}^{2}P_{2}^{2}P_{3}^{2}\right) \end{array}$$

where *A*_1_ and *A*_11_ are landau coefficients depending on local strain field concentration (*c*) and temperature (*T*). *A*_111_, *A*_112_, *A*_113_ are constant coefficients. Additionally, the landau coefficients *A*_12_ not only depend on *c* and *T* but also relate to *E*_ba_/*E* which represents the energy barrier of polar rotation. And the tricritical point is established near *c* = 0.03. The gradient free energy representing the energy from polarization inhomogeneity is described as $f_{\mathrm{grad}}=\frac{1}{2}G_{11}\left(\sum_{i=1,2,3;j=1,2,3}{\left(P_{i,j}\right)}^{2}\right)$, where *G*_11_ is the gradient energy coefficient. The long‐range elastic strain energy can be written as: $f_{\mathrm{elas}+\mathrm{localelas}}=\frac{1}{2}C_{ijkl}e_{ij}e_{kl}=\frac{1}{2}C_{ijkl}\left(\varepsilon_{ij}-\varepsilon_{ij}^{0}+\varepsilon_{ij}^{\mathrm{local}}\right)\left(\varepsilon_{kl}-\varepsilon_{kl}^{0}+\varepsilon_{kl}^{\mathrm{local}}\right)$, where *C_ijkl_* is the elastic stiffness tensor, *e_ij_*, *ε_ij_*, *ε* ^0^ *_kl_* and *ε* ^local^ *_kl_* are the elastic strain, total strain, electrostrictive stress‐free strain and local strain from experimental lattice distortion beside spontaneous strain. And *ε* ^0^ *_kl_* = *Q_ijkl_P_i_P_j_*, where *Q_ijkl_* is the electrostrictive coefficient. The steps for obtaining local strain field are as following: “Firstly, the high‐resolution image is analyzed by using geometrical phase analysis (GPA) to obtain the maps of different strain components (*ε_xx_*, *ε_xy_* and *ε_yy_*). Secondly, the maps of different strain components are correspondingly gridded by using interpolation functions to obtain the data of total strain matrix. Thirdly, the tensor of local strain field (*ε* ^local^) equals the total strain matrix minus the spontaneous strain. The long‐range electrostatic energy is calculated by the following equation: $f_{\mathrm{elec}}=-\frac{1}{2}E_{i}P_{i}-\frac{1}{2}E_{i,\mathrm{depol}}\bar{P_{i}}-E_{i,\mathrm{appl}}\bar{P_{i}}$, where *E_i_* is the inhomogeneous electric field due to the dipole‐dipole interactions, *E_i,_*_depol_ is the average depolarization field due to the presence of surface charges, *E_i,_*_appl_ is the external electric field applied in the *i*th direction, $\bar{P_{i}}$ is the average polarization. The local random distribution electric field effect related with dopant valence and concentration is described as:$f_{\mathrm{localelec}}=E_{i,\mathrm{localelec}}\bar{P_{i}}$, where *E_i,_*_localelec_ is the random distribution electric field. The temporal evolution of the domain structure can be obtained by solving the time‐dependent Ginzburg‐Landau (TDGL) equation:{*dP_i_* (*x*,*t*)}/*dt* = −*M*{δ*F*/δ*P_i_* (*x*,*t*)}, *i* = 1, 2, 3, where *M* is the kinetic coefficient related to the domain mobility and *t* is time.

To describe the critical phase transition state, an isotropic state is assumed at tricritical point composition and temperature in our simulations, and related parameters used in the calculations are as follows:^[^ ^71^ ^]^ landau coefficients $A_{1}=A_{1}^{0}(T-T_{C}^{00})$, $A_{11}=A_{11}^{00}\left|c-c_{tr}\right|$, $A_{111}=A_{111}^{0}$, $A_{12}=A_{12}^{0}\left(T-T_{tr}\right)+A_{12}^{00}(c-c_{tr})$, $A_{112}=A_{112}^{0}$ and $A_{113}=A_{113}^{0}$, where $A_{1}^{0}=4.124\times 10^{5}$, $A_{11}^{00}=-4\times 10^{8}$, $A_{12}^{0}=\frac{E_{ba}}{E}\times 5\times 10^{6}$, $A_{12}^{00}=\frac{E_{ba}}{E}\times 128\times 10^{8}$, $A_{111}^{0}=1.294\times 10^{9}$, $A_{112}^{0}=3.882\times 10^{9}$, $A_{113}^{0}=7.764\times 10^{9}$ (all in SI units), *c_tr_* = 0.03, *T_tr_* = 303 K, $T_{C}^{00}=343K$; elastic constants *C*_11_ = 1.78 × 10^11^Nm^−2^, *C*_12_ = 9.64 × 10^10^Nm^−2^, and *C*_44_ = 1.22 × 10^11^Nm^−2^; electrostrictive coefficients of BaTiO_3_ *Q*_11_ = 0.11C^−2^ · m^4^, *Q*_12_ = −0.045C^−2^ · m^4^, and *Q*_44_ = 0.059C^−2^ · m^4^.^[^ ^72^ ^]^ The domain wall energy for 90° domain walls is around 0.1 J m^−2^, which yields a length scale *l*_0_ (the numerical grid size) of ≈2.0 nm. The simulations were carried out in two dimensions with cell sizes 256×256 grids with periodic boundary conditions.

## Conflict of Interest

The authors declare no conflict of interest.

## Supporting information



Supporting Information

## Data Availability

The data that support the findings of this study are available from the corresponding author upon reasonable request.
