# Supplementary material for: Boosts in Polarization and Piezoelectric Responses of Lead‐Free Ferroelectrics through Strain‐Enhanced Glassy Coexistent Polars with High Dynamics
Source: Adv Sci (Weinh). 2025 Jul 11;12(37):e02973. doi: 10.1002/advs.202502973 (PMC12499439; doi:10.1002/advs.202502973)
Supplement: Supplementary file 1 — Supporting Information [file ADVS-12-e02973-s001.docx]

**Supplemental Material**

**for**

**Boosts in Polarization and Piezoelectric Responses of Lead-Free Ferroelectrics through Strain-Enhanced Glassy Coexistent Polars with High Dynamics**

Liqiang He^1^, Le Zhang^1,4^*, Yating Ran^2,5^, Zibin Chen^2,5^*, Chuanxin Liang^1^, Yanshuang Hao^3^, Jianwei Li^1^, Zhizhi Xu^1^, Sen Yang^1^, Michael A. Carpenter^4^, Xiaobing Ren^1,3^ and Dong Wang^1^*

1. Frontier Institute of Science and Technology and School of Physics, State Key Laboratory for Mechanical Behavior of Materials and MOE Key Laboratory for Non-equilibrium Synthesis and Modulation of Condensed Matter, Xi’an Jiaotong University, Xi’an, Shaanxi, 710049, China
2. State Key Laboratory of Ultra-precision Machining Technology, Department of Industrial and Systems Engineering, The Hong Kong Polytechnic University, Hong Kong, China
3. Center for Functional Materials, National Institute for Materials Science, 1-2-1 Sengen, Tsukuba, 305-0047, Ibaraki, Japan
4. Department of Earth Sciences, University of Cambridge, Cambridge, CB2 3EQ, UK
5. Research Institute for Advanced Manufacturing, Department of Industrial and Systems Engineering, The Hong Kong Polytechnic University, Hong Kong, China

**Determination of Burns temperature (*T*_B_) and freezing temperature (*T*_f_)**

The Burns Temperature (*T*_B_), at which the ergodic polar nano region appears in the paraelectric phase, can be determined by the temperature where the dielectric permittivity deviates from the Curie-Weiss law [1]:

$$\varepsilon=\frac{C}{T-T_{CW}}$$

where *ε* is dielectric permittivity, *C* the Curie-Weiss constant and *T*_CW_ the Curie-Weiss temperature.

Upon cooling to the static freezing temperature (*T*_f_), the polar nano regions (PNRs) in the ergodic state become frozen into a non-ergodic state. The relation of the frequency/temperature can be described by the following Vogel-Fulcher relation [2,3]:

$f=f_{0}e^{\frac{-E_{a}/k}{T_{\max}-T_{f}}}$ → $\ln f=\ln f_{0}+\frac{-E_{a}/k}{T_{\max}-T_{f}}$

where *T*_f_ is the static freezing temperature/K, *E*_a_ the activation energy/eV, k is the Boltzmann constant, *f*_0_ the Debye frequency/Hz and *T*_m_ the temperature of the maximum dielectric permittivity/K.


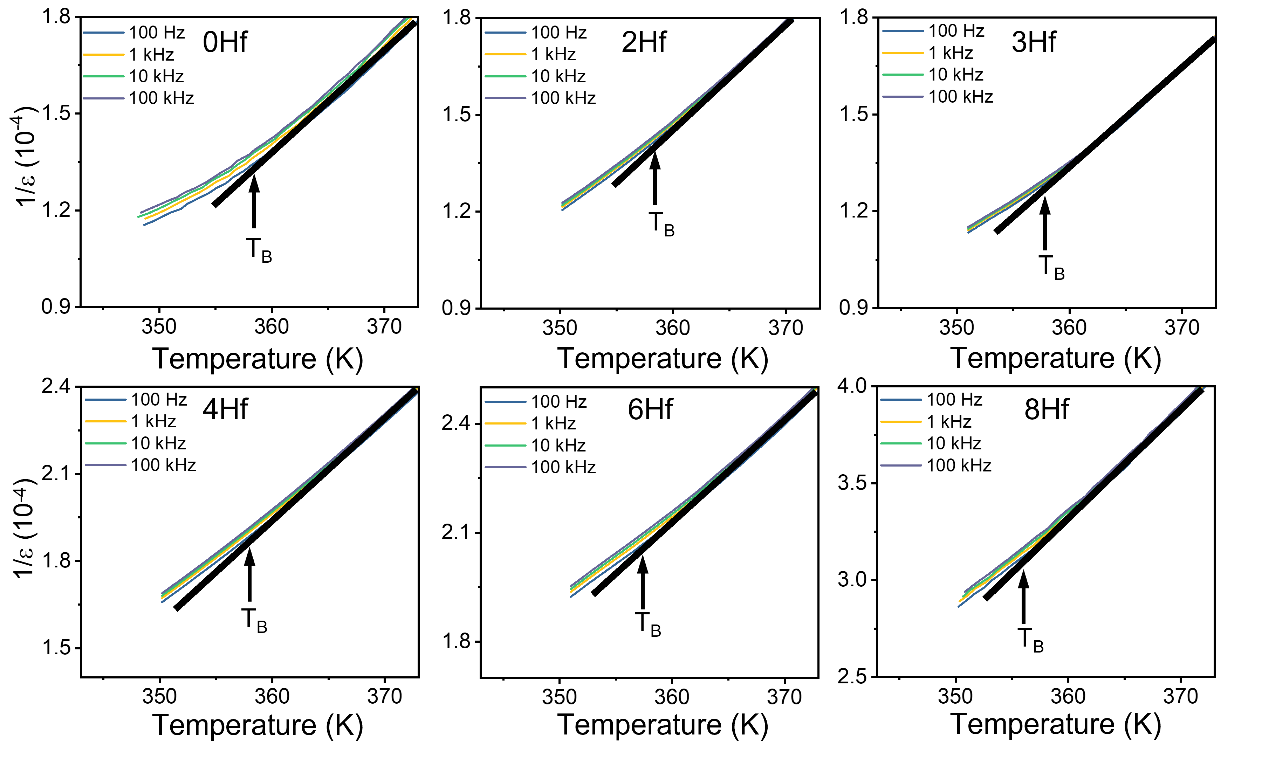


**Figure S1.** Curie-Weiss fitting results for the temperature-dependent frequency dispersion of reciprocal dielectric permittivity (1/*ε*) of (Ba_0.84_Ca_0.16_)_0.985_Bi_0.01_(Ti_0.9_Zr_0.07_Sn_0.03_)_1-_*_x_*_/100_Hf*_x_*_/100_O_3_ (*x*Hf, *x*=0, 2, 3, 4, 6 and 8) ceramics, well giving the information of *T*_B_ in the phase diagram.


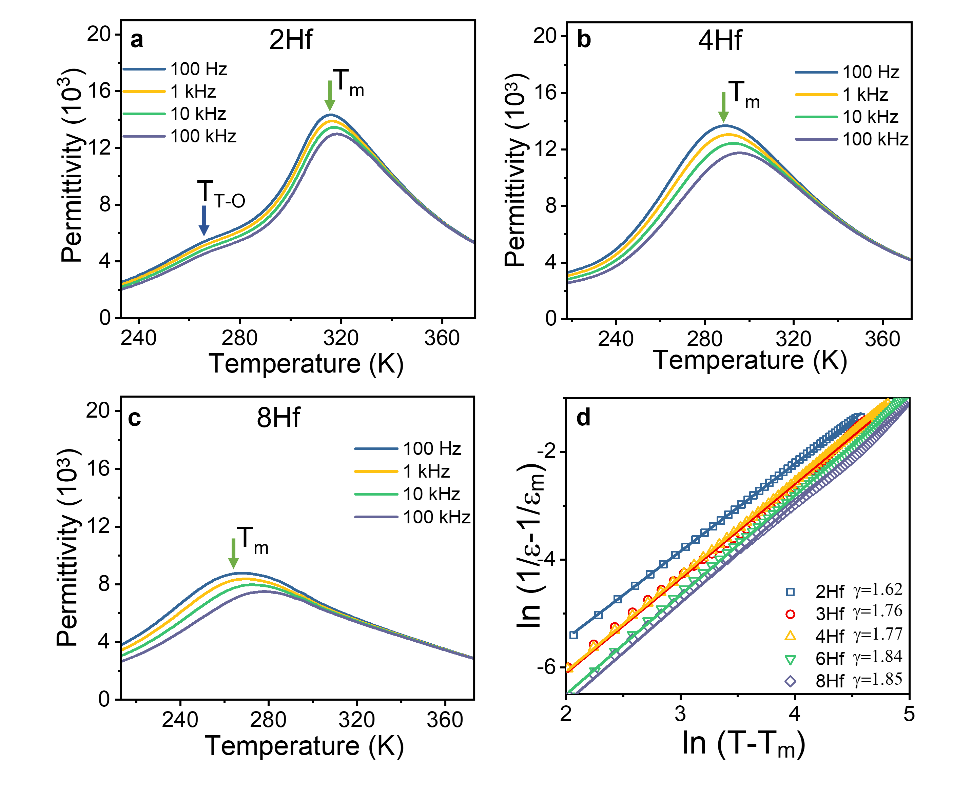


**Figure S2.** The temperature-dependent dielectric permittivity (*ε*) curves of (a) 2Hf, (b) 4Hf and (c) 8Hf under different frequencies respectively. (d) Temperature-dependent dielectric permittivity fitted by the modified Curie-Weiss relation under 100 Hz, showing the increased diffuseness with the increment of Hf concentration, suggesting strengthened relaxation process of nanodomains.


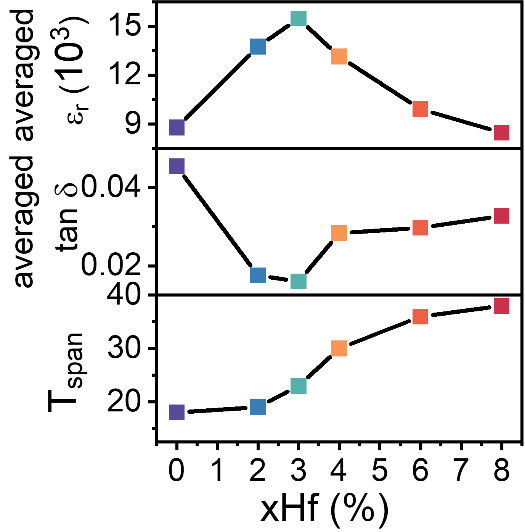


**Figure S3.** Composition-dependent averaged *ε*_r_, averaged tan *δ* and thermal stability (*T*_span_) of *x*Hf (*x*=0, 2, 3, 4, 6 and 8) ceramics.


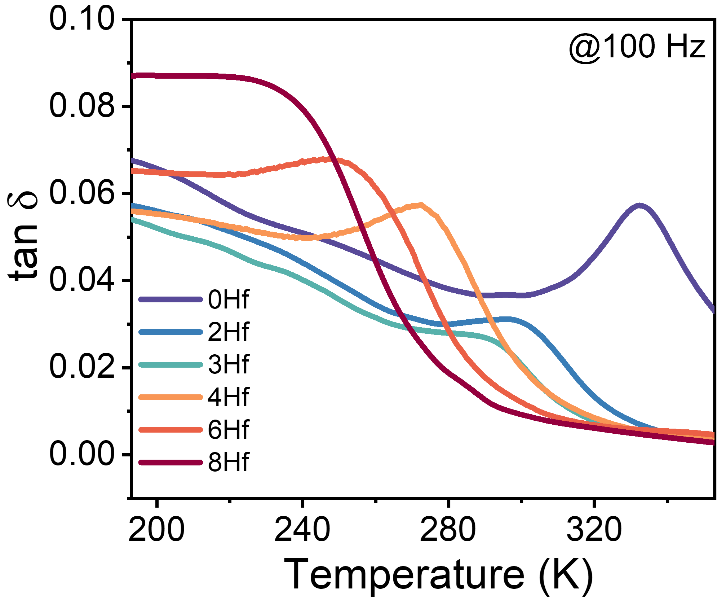


**Figure S4.** The temperature-dependent dielectric loss (tan *δ*, *δ* is the phase difference between current and voltage) of *x*Hf (*x*=0, 2, 3, 4, 6 and 8) ceramics under 100 Hz.


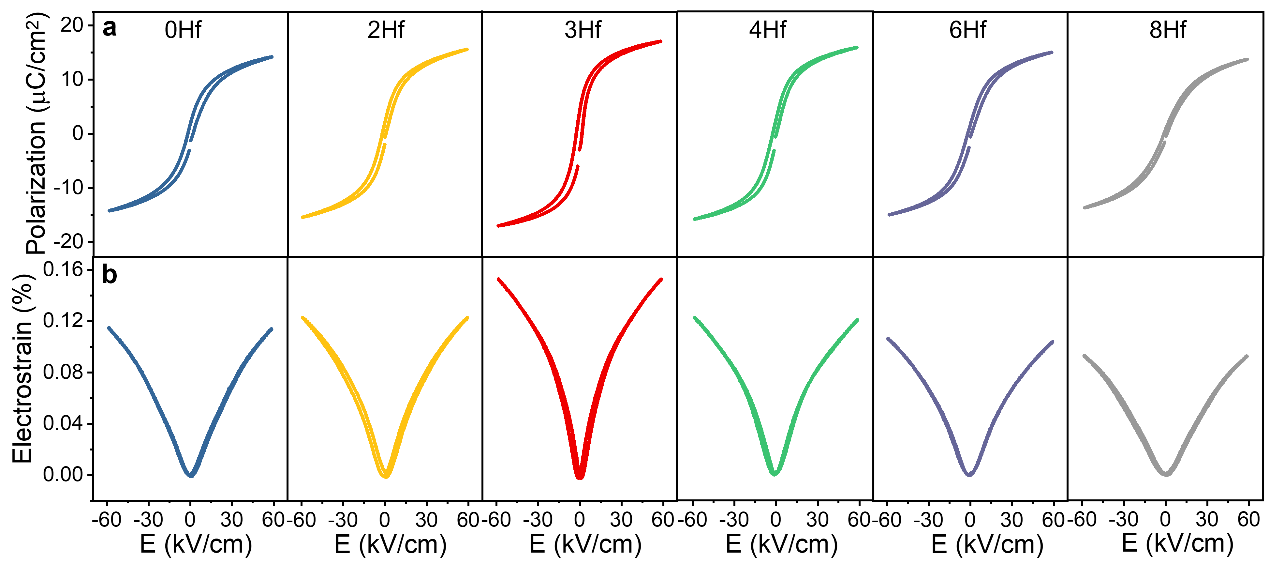


**Figure S5.** (a) Polarization (*P*) and (b) electrostrain (*S*) versus electric field (*E*) curves of *x*Hf (*x*=0, 2, 3, 4, 6 and 8) ceramics at *T*_m_ under the drive field of 60kV/cm.

**
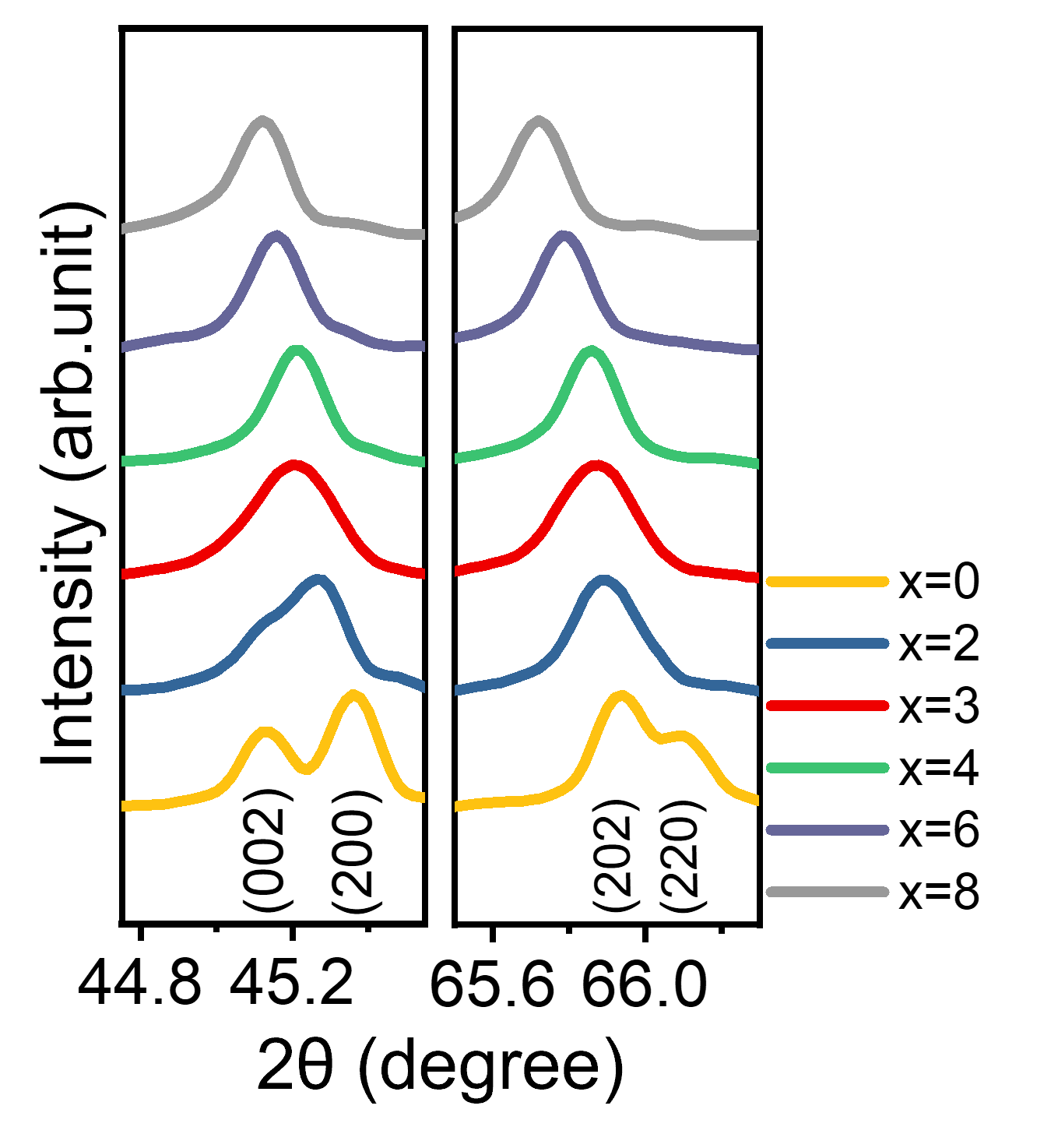
**

**Figure S6.** The characteristic peaks of (002)/(200) and (202)/(220) .

**
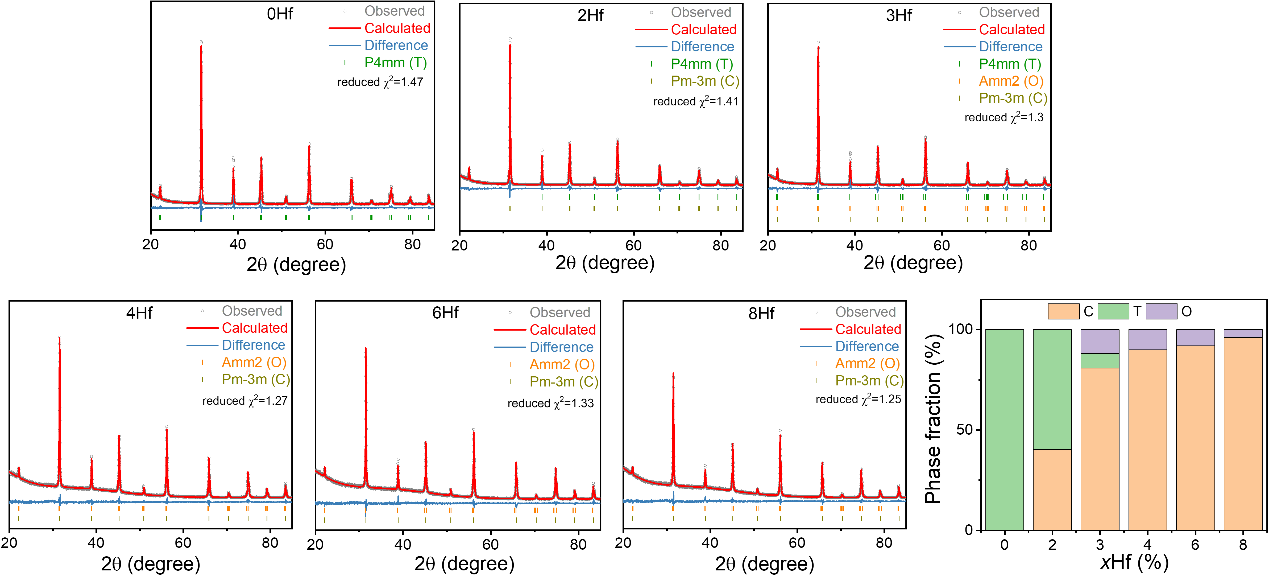
**

**Figure S7.** Rietveld refinement results for the XRD data and evolution of room-temperature phase fraction of *x*Hf (*x*=0, 2, 3, 4, 6 and 8) ceramics.


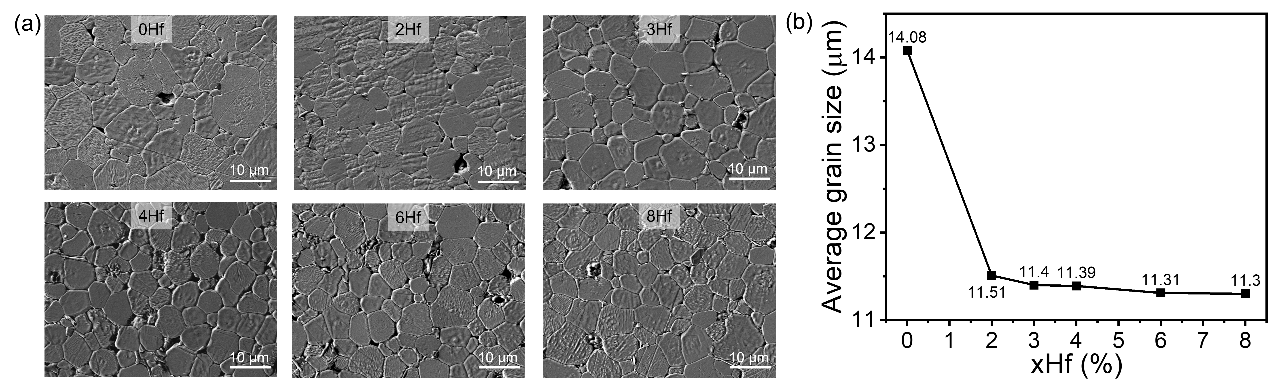


**Figure S8.** (a) SEM morphology images and (b) average grain size of *x*Hf (*x*=0, 2, 3, 4, 6 and 8) ceramics. The samples are polished and then thermally etched at 1473 K for 30 minutes before testing.


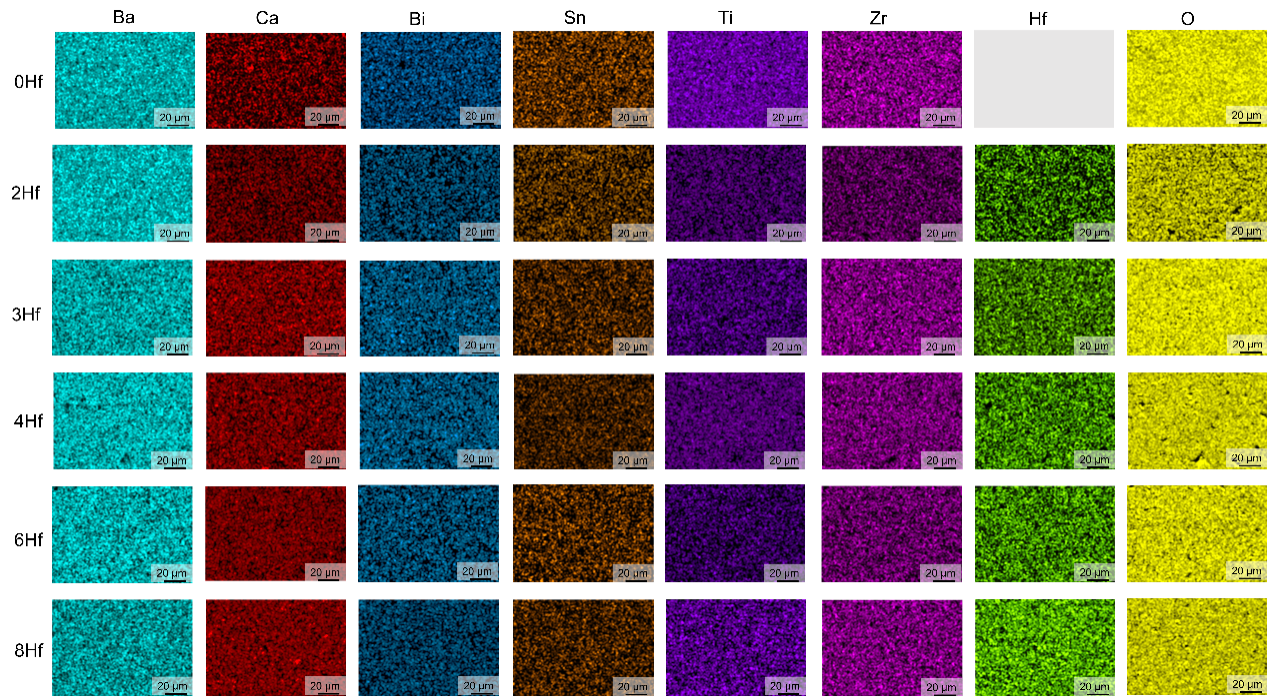


**Figure S9.** SEM-EDX mapping images for each element of *x*Hf (*x*=0, 2, 3, 4, 6 and 8) ceramics.


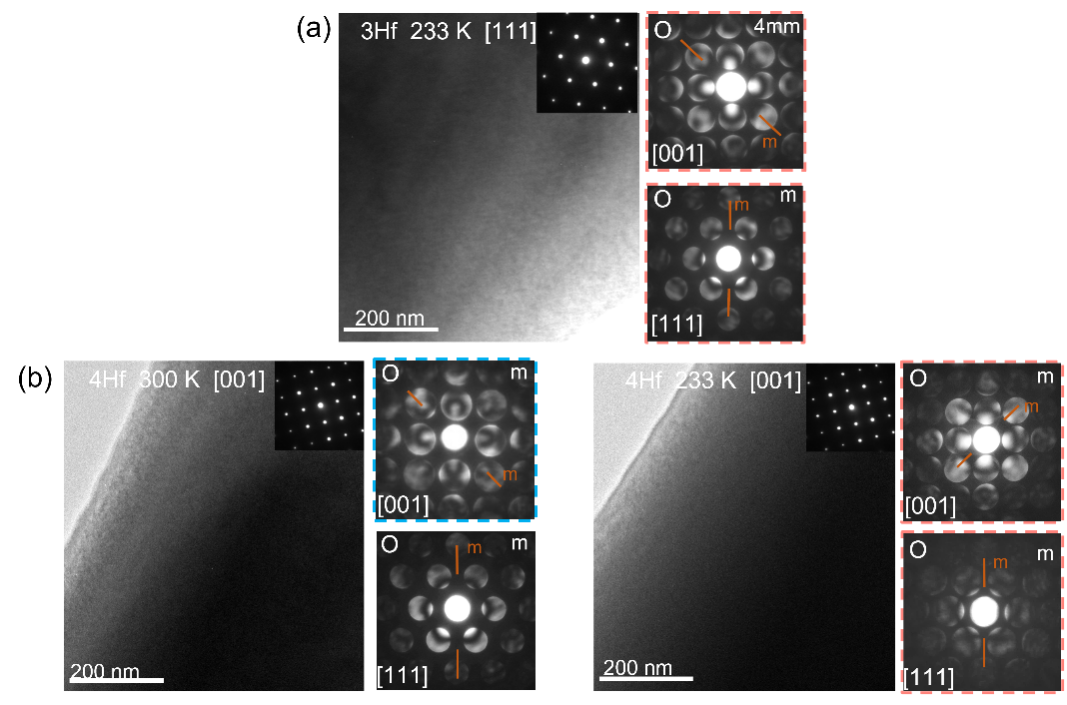


**Figure S10.** (a) Bright field images and convergent beam electron diffraction (CBED) patterns along [001] and [111] zone axes of 3Hf at 233 K. (b) Bright field images and CBED patterns along the incidence axis of [001] and [111] of 4Hf at 300 K and 233 K.


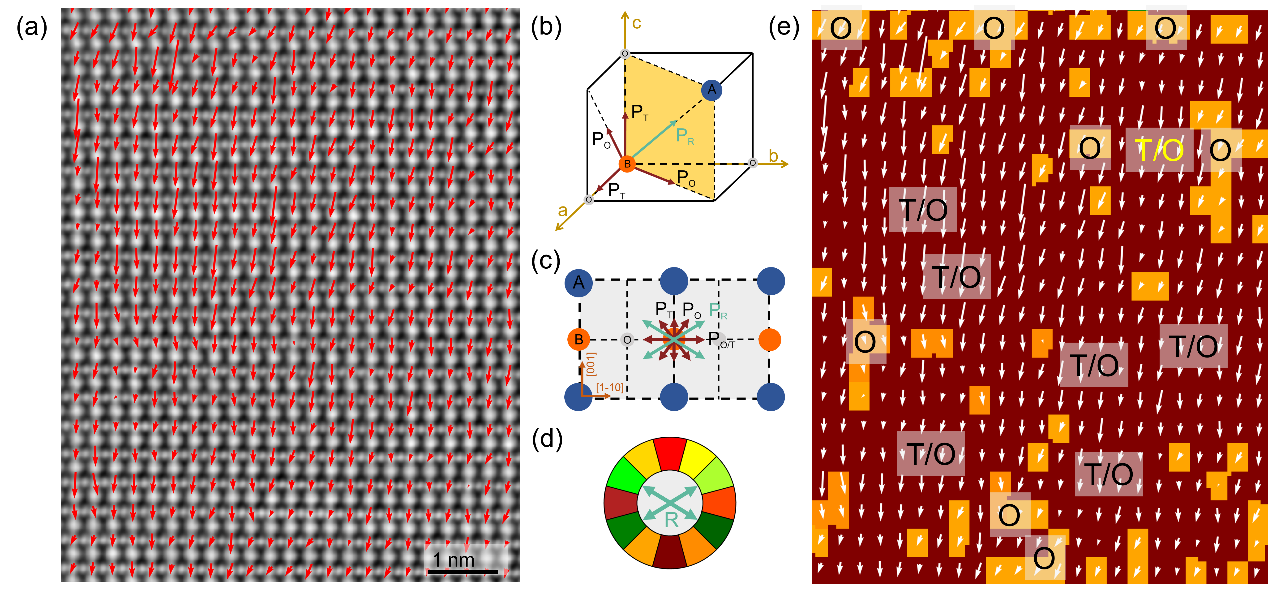


**Figure S11.** (a) Atomic-resolution iDPC-STEM polarization vector image along [110] zone axis. (b) Schematic figure of polarization directions for Tetragonal (T), Orthorhombic (O), and Rhombohedral (R) in the ABO_3_ structure. (c) Schematic projection along the [110] zone axis, showing polarization directions of T, O and R. (d) Colors used for distinguishing the polarization directions. (e) Polarization angle mapping with the distribution of T and O phases, showing the absence of R phase.


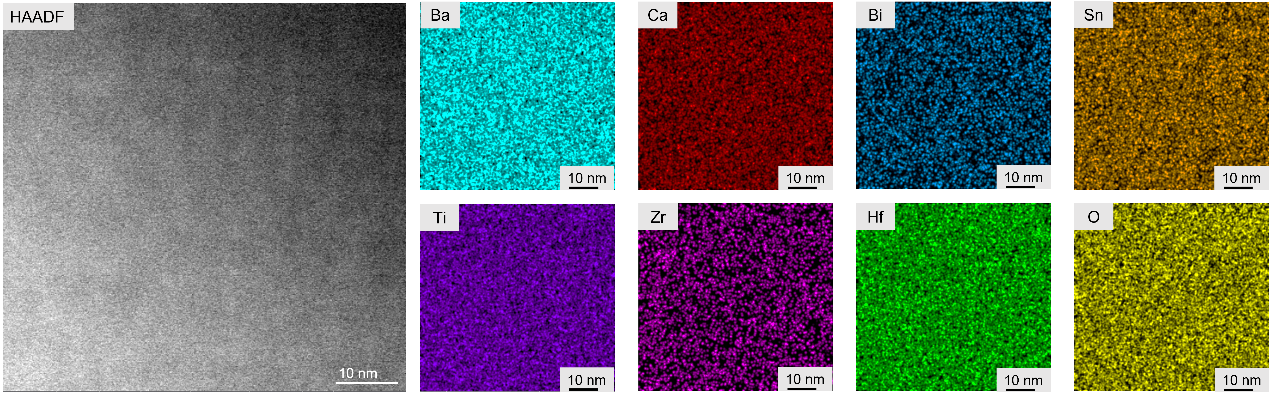


**Figure S12.** HAADF-STEM image and localized energy-dispersive X-ray spectroscopy mappings for each element of 3Hf ceramics along [110] zone axis.


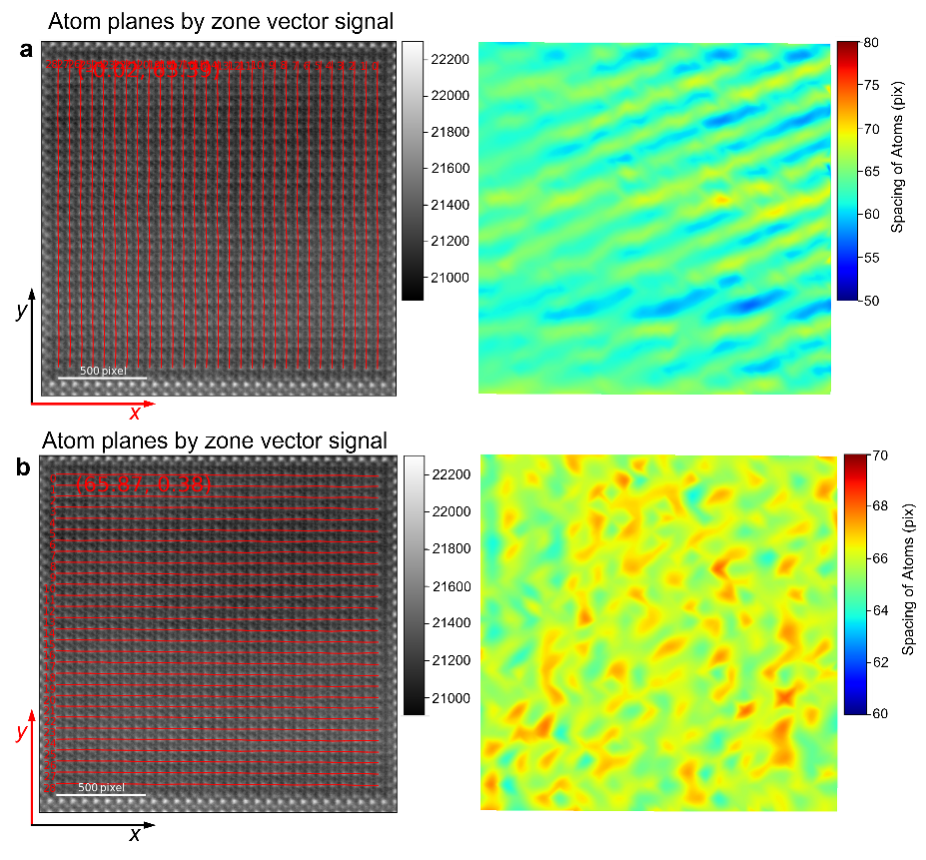


**Figure S13.** B-site atom planes by zone vector signal and the related spacing mapping of atoms along (a) *x* and (b) *y* directions, where lattice distortion *ε_xx_*= (spacing mapping of B-site atoms along *x*/63.39)-1, *ε_yy_*= (spacing mapping of B-site atoms along *y*/65.87)-1 and *ε_xy_*= the ratio of space mapping of B-site atoms-1.


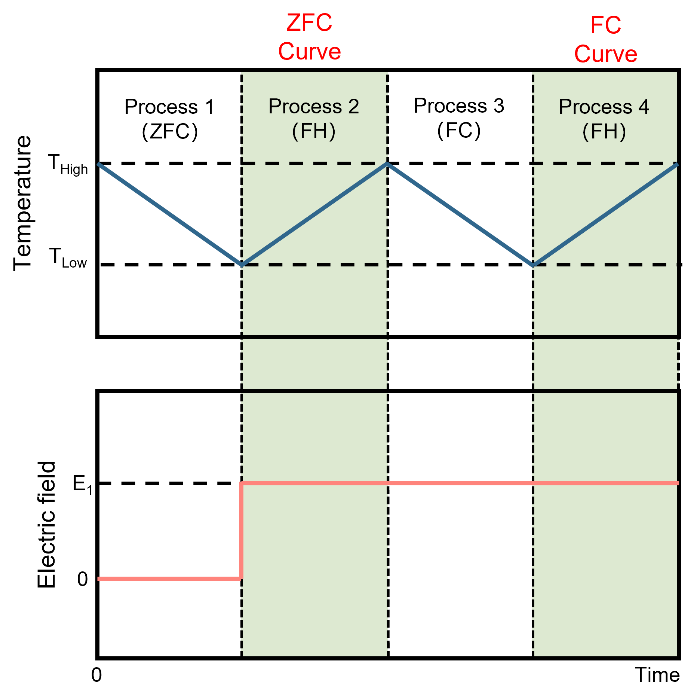


**Figure S14.** Schematic calculated procedure of the zero-field-cooling (ZFC)/field-cooling (FC) measurement for ferroelectric glass, revealing the nonergodicity of a glass system. The glassy ferroelectric system is first cooled to a temperature (*T*_Low_) well below the freezing temperature (*T*_f_) under zero electric field (ZFC, process 1). Then, an external electric field (*E*_1_) is applied and the system is heated to a temperature (*T*_High_) far above *T*_f_ (FH, process 2). Thereafter, the system is cooled to *T*_Low_ again at the same electric field (FC, process 3) and then heated to *T*_High_ again at the same electric field (FH, process 4). The static polarization curves that calculated in process 2 and process 4 are called the ZFC curve and FC curve, respectively. And their deviation is a signature for nonergodicity [4].


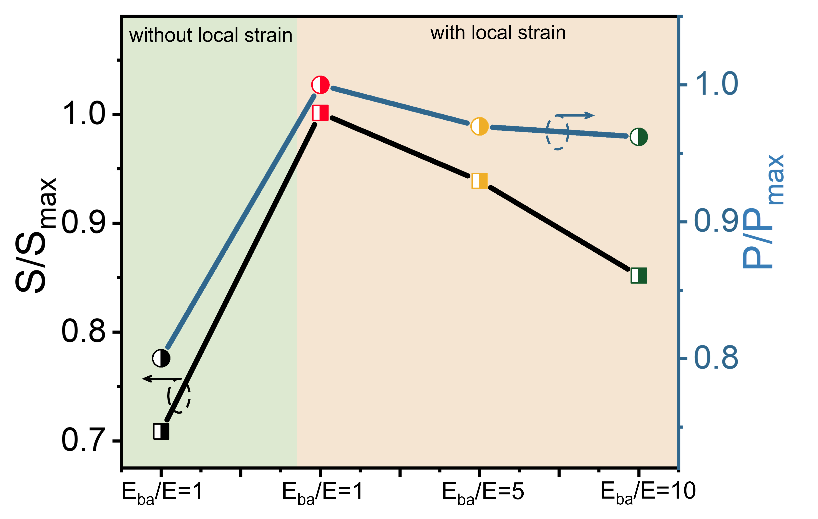


**Figure S15.** The comparison of electrostrain and maximum polarization values under the same external electric field when changing the strain field and *E*_ba_/*E* conditions.


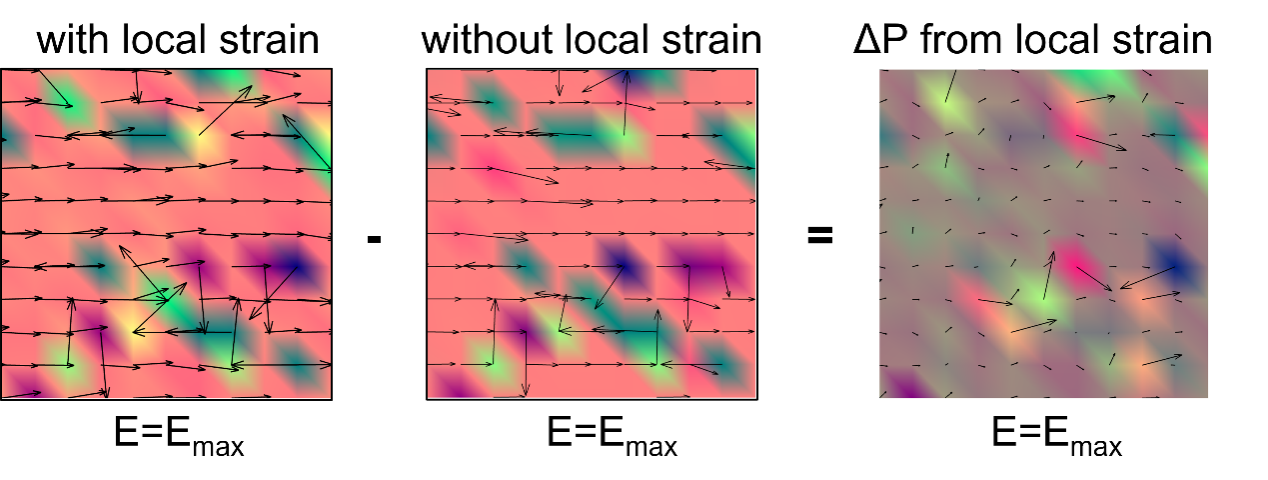


**Figure S16.** Polarization enhancement (ΔP) map calculated by subtracting polarization vectors without local strain from those with local strain at the same condition of *E*_ba_/*E* =1 and under the same maximum electric field (*E*_max_).


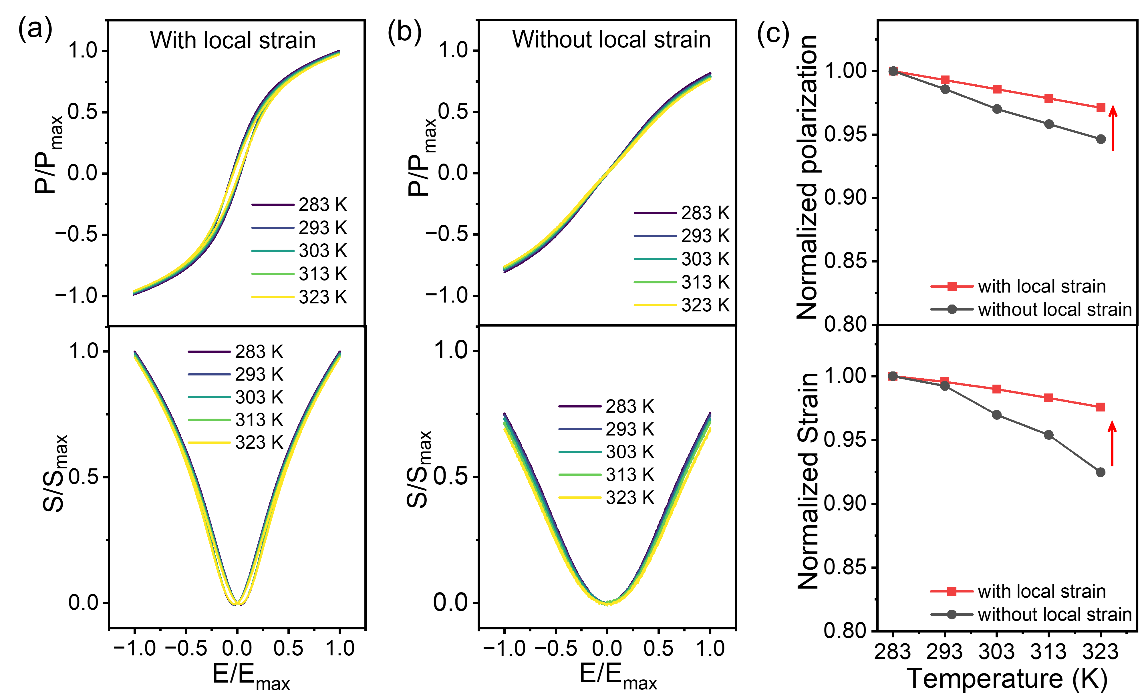


**Figure S17.** Calculated temperature-dependent polarization (*P*) and electrostrain (*S*) versus electric field (*E*) loops of two cases. (a) with local strain and (b) without local strain. (c) The evolution of normalized maximum polarization and electrostrain upon heating from 283 K to 323 K.

**Table S1.** Comparison of *ε_r_*, tan *δ*, *ε*_r_/tan*δ* and *T*_span_ of our 3Hf ceramic with other representative ferroelectric and relaxor ceramics including Hf-doped BaTiO_3_-based ceramic systems.

| Materials | *ε*_r_ | tan *δ* | *ε*_r_/tan*δ* (10^5^) | *T*_span_ (K) |
| --- | --- | --- | --- | --- |
| BT-30Hf^[49]^ | 2958.84 | 0.075 | 0.40 | 33 |
| BTSAB-0.3H^[50]^ | 212.5 | 0.032 | 0.07 | 122 |
| BaHf_0.1_Ti_0.9_O_3_^[51]^ | 7534.7 | 0.014 | 5.38 | 22 |
| BT-0.11Hf^[52]^ | 15541 | 0.04 | 3.89 | 11 |
| Ba(Hf_0.08_Ti_0.92_)O_3_^[53]^ | 17094 | 0.036 | 4.75 | 11 |
| BHT-1^[54]^ | 6682 | 0.011 | 6.07 | 21 |
| BCZHT^[55]^ | 3461 | 0.051 | 0.68 | 22 |
| BHT10^[56]^ | 17173 | 0.02 | 8.59 | 7 |
| BZHT^[57]^ | 14100 | 0.01 | 14.10 | 15 |
| BT-CH^[38]^ | 21416 | 0.035 | 6.12 | 8 |
| KNN^[42]^ | 6323 | 0.072 | 0.87819 | 5 |
| BTS10.5^[40]^ | 53644 | 0.07 | 7.66342 | 4.5 |
| BCZT^[39]^ | 16629 | 0.02 | 8.3145 | 8 |
| BZT-BCT-Mn^[41]^ | 8428 | 0.02 | 4.214 | 10 |
| BLZT^[44]^ | 8551 | 0.09 | 0.95011 | 18 |
| KNN-BSZ^[43]^ | 10000 | 0.05 | 2 | 20 |
| PMN^[45]^ | 8324 | 0.12 | 0.69367 | 32.6 |
| BCTS12^[36]^ | 17000 | 0.05 | 3.4 | 34 |
| BMT-PMN-PT^[46]^ | 11738 | 0.075 | 1.56507 | 38 |
| X7R^[48]^ | 3000 | 0.01 | 3 | 43 |
| BBiT^[47]^ | 2447 | 0.1 | 0.2447 | 59 |
| 3Hf (this work) | 15800 | 0.016 | 9.88 | 23 |

**Table S2.** Fitting *T*_f_, *E*_a_, *f*_0_ and Adj. R-Square (R^2^) of *x*Hf (*x*=2, 3, 4, 6 and 8) on the basis of Vogel-Fulcher relation.

|  | 2Hf | 3Hf | 4Hf | 6Hf | 8Hf |
| --- | --- | --- | --- | --- | --- |
| *T*_f_ (K) | 313.8 | 300.6 | 283.6 | 268.7 | 253.6 |
| *E*_a_ (meV) | 0.61 | 0.96 | 3.36 | 3.82 | 3.80 |
| *f*_0_ (10^7^ Hz) | 0.80 | 1.48 | 0.83 | 0.78 | 0.51 |
| R^2^ | 0.98543 | 0.98171 | 0.98266 | 0.98673 | 0.98963 |

**Supplementary references**

[1] A. A. Bokov and Z. -G. Ye, *J. Mater. Sci.* **2006**, *41*, 31.

[2] A. E. Glazounov and A. K. Tagantsev, *Appl. Phys. Lett.* **1998**, *73*, 856.

[3] D. Viehland, S. J. Jang, L. E. Cross and M. Wuttig, *J. Appl. Phys.* **1990**, *68*, 2916.

[4] Y. Wang, X. Ren, K. Otsuka, and A. Saxena, *Phys. Rev. B* **2007**, *76*, 132201.

-
